# Exploring the Lived Experiences of Caregiving for Older Family Members by Young Caregivers in Singapore: Transition, Trials, and Tribulations

**DOI:** 10.3390/ijerph21020182

**Published:** 2024-02-05

**Authors:** Araviinthansai Subramaniam, Kalyani Kirtikar Mehta

**Affiliations:** S R Nathan School of Human Development, Singapore University of Social Sciences, Singapore 599494, Singapore; kskm2013@gmail.com

**Keywords:** young carers, young adult carers, young caregivers, family, caregiving, informal care, intergenerational relationships, Singapore, aging, IPA

## Abstract

Amidst population ageing trends and epidemiological transitions, there has been a growing emergence of young family caregivers, about whom most studies have been conducted in Western countries. Their subjective experiences and perceptions toward caregiving remain underexplored in Asia. This qualitative study explored the lived experiences of caregiving for older family members by young caregivers in Singapore. Interpretative phenomenological analysis was employed to collect and analyse data from semi-structured, in-depth interviews with six young adult caregivers aged between 23 and 29. Interviews were supplemented with photo-elicitation techniques to deepen interview discussions and uncover experiential significance. Findings illustrated transitions into caregiving, challenges across role conflicts and expectations amidst developmental tasks and transitions, and navigation of intergenerational conflicts and ambivalence. Although no definitive conclusions can be reached from this small-scale study, the findings offer important insights into the convergence and intensity of young caregivers’ experiences. Given that caregiving challenges are likely to continue amidst Singapore’s rapidly ageing population, these necessitate further in-depth research efforts. Implications for policy and practice across multiple stakeholders interfacing with youth and older adults are presented. A whole-of-society approach is called for to enable young caregivers to realise their full potential while contributing to their ageing families and nation.

## 1. Introduction

Greater longevity and a declining fertility rate are causing the global population to age rapidly, with an estimated 101 million older adults aged 60 and above dependent on family members for their care [1]. The Eastern and Southeastern regions of Asia will witness the largest increase in the proportion of older persons, with Singapore being one of the fastest-ageing nations globally and in the region [2]. By 2030, 1 in 4 citizens in Singapore will be aged 65 and above [3]. By 2050, 1 in 2 of the total population will be aged 65 and above [4]. Such a profound demographic transition is accompanied by a growing proportion of seniors with multimorbidity [5], with 1 in 2 healthy residents aged 65 and above potentially becoming severely disabled in their lifetime, which will prolong for ten years or more for 3 in 10 seniors [6]. These would intensify the demand for long-term care from family members of older adults.

Yet, with shrinking family sizes, the old-age support ratio will decline from 3.8 in 2022 to 2.7 by 2030. This is against the backdrop of socio-demographic undercurrents such as plummeting fertility rates and later parenthood, alongside a significant rise in the old-age population amidst extended life expectancy [3,7,8]. These induce verticalisation and horizontal narrowing of family structures, resulting in “longer years of shared lives between generations”, with prolonged caregiving relationships [9]. This increases the potential for caregiving by younger generations for older parents and surviving grandparents. 

Consequently, Singapore has observed a rising number of young caregivers for older family members [10]. According to the Agency for Integrated Care (AIC), which coordinates aged care services in Singapore, the number of caregivers under 35 who tapped into the Caregivers Training Grant grew from 287 in 2013 to 465 in 2017. Some social service agencies (SSAs) report witnessing significant numbers of young caregivers, attributed partly to the earlier onset of diseases, which were once construed to be prevalent in old age. Dementia Singapore (formerly Alzheimer’s Disease Association) reported that annually, up to 200 young individuals are thrust into the role of caregiving amidst the rising trend of adults contracting dementia earlier, with caregivers being as young as 12 years old [11,12]. However, there is currently no nationally representative research on the prevalence of young caregivers among older adults or dedicated exploratory local studies on their experiences and impact of caregiving. Thus, practitioners and policymakers have limited data to draw upon to inform the formulation of targeted support.

While young caregivers in the Western world are increasingly being studied and recognised as a distinct social group alongside varying levels of policies [13], there remains a notable gap, especially in Asia, in exploring the experiences of children and young adults in family caregiving roles, despite them existing in every country [14,15]. This is complexified by the lack of self-awareness among young caregivers as being family caregivers amidst cultural expectations around care, alongside caregiving being typified as a mid-life or old-age phenomenon by societal norms [16,17]. Therefore, young caregivers remain an overlooked, invisible population, falling through the gaps of public policy and practice amid the discrete boundaries of health, social care, and education services and within the wider caregiving literature [18,19].

Notably, young adult caregivers are experiencing critical developmental tasks and life transitions such as identity formation, growing autonomy, navigating intimate relationships, tertiary education, career development, leaving the parental home, and starting a family [20,21]. The demands of caregiving may challenge the negotiation of these milestones, with resultant implications for overall well-being [22,23]. Becker and Becker [24] (p. 6) defined “young adult carers” in the United Kingdom (UK) as individuals aged 18–24 “who provide or intend to provide care, assistance, or support to another family member on an unpaid basis.” However, extant literature addressing young adult caregivers utilises competing age-based definitions of young adulthood, ranging from 24–25 years old [25,26], to 29 years of age [27,28], or to a more expansive age threshold of 40 years old [29].

These expanded age bands are attributable to the prolonged life course period of young adulthood over the last decades, due to later average ages of life-stage transitions (e.g., transition to a stable career, marriage, parenthood), especially within developed nations [30]. These trends are consistent within Singapore, with the age definition of youths being 15–35 [7,31]. Hence, developmental theorists assert that young adults reside at a qualitatively unique life stage, which impacts the nature of caregiving experiences, outcomes, and needed support services [32]. 

Young caregivers assume varied responsibilities, from provisioning emotional support and companionship to assisting with basic and instrumental activities of daily living (ADL) for older family members. To this end, the widely documented effects of caregiving on well-being among adult family caregivers may even be more pertinent to young caregivers yet distinctively experienced owing to their life stage [33,34].

Caregiver burden is one of the primarily investigated outcome indicators for the multifaceted strains and responses associated with physical, psychological, social, and financial demands and challenges arising from caregiving, including impaired family relationships [35,36,37]. In this regard, caregiver burden is related to the well-being of the care recipient, caregiver, and the wider family system. While caregiver burden is not confined to any specific culture and has been well-established in the adult caregiver literature, there are distinctive elements that bear an influence on the Asian caregiving experience. Given that family assumes precedence in Asian societies like Singapore, buttressed by values and expectations of filial piety and responsibility [38,39,40], it is imperative to study how caregiver burden is experienced among Asian young caregivers as well. Among the dimensions of caregiver burden, psychological dysfunctions such as depression and anxiety remain the most enduring and widely studied forms among adult caregivers [41,42,43].

In Singapore, based on the 2018 Quality of Life (QOL) of caregivers study by the National Council of Social Service (NCSS) [44], caregivers of both older and younger individuals with disabilities, chronic illnesses, mental health conditions, or frailty reported lower quality of life, especially in the domains of personal belief and social relationships. Amongst other predictors, caregivers co-residing with care recipients, caring for more than one individual, and engaging in higher duration and intensity of care reported significantly lower quality of life. Further, according to the 2013 Well-being of the Singapore Elderly (WiSE) population-based cross-sectional survey, a sizeable 24.5% of all caregivers experienced burden, whereas 46% encountered distress from care recipients’ behavioural and psychological symptoms of dementia and 8.8% underwent psychiatric morbidity [45,46]. Similarly, based on the Singapore Survey on Informal Caregiving (2010–2011), the leading predictor of caregivers’ depressive symptomatology was their negative reaction to caregiving. This arose from weak family support, apart from the care recipient’s health and functional and cognitive limitations, which intensified caregivers’ negative reactions [47].

Although these local studies provide valuable indications of the multifaceted strains associated with caregiver burden, they failed to report disaggregated findings on the youngest age band owing to methodological constraints (e.g., small sample size) and excluded caregivers aged 18 and below. As the samples were heterogeneous in age, amalgamating young adult caregivers with older cohorts, it was beyond the scope of these studies to investigate young caregivers’ specific experiences. Hence, they provide limited contextual and targeted insights to design and implement services attuned to the needs of young caregivers. Bastawrous [36] further contends that quantitative measures of caregiver burden fail to capture critical contextual elements of the caregiving experience. They preclude the nuanced subjectivity and sophistication inherent to human experiences within sociocultural and familial contexts, which instead warrant qualitative explorations. 

This is pertinent given Singapore’s socio-political stance, which maintains that families remain the first line of support. Fostering intergenerational support constitutes a core component of the city-state’s ageing policies in areas across legislation, housing, taxation, health care, and financial subsidies [48], while promoting ageing in place that is commensurate with the preferences of older adults [49]. This reflects the wider socio-cultural norms within Asian families, which buttress expectations for intergenerational interdependence across the life course [50]. 

Familial obligations are reinforced by the presiding culture of filial piety in Singapore [51], which is also evident among the youths. According to the National Youth Council [52], youths have consistently ranked maintaining strong family ties as a top life goal over the past decade. In 2019, 8 in 10 youths reported they would care for parents in old age irrespective of circumstances, reflecting the propensity for caregiving underpinned by the pertinence of family values among young individuals. Hence, although it is posited that nations with underdeveloped long-term care systems may have a larger incidence of young caregivers [53], the duty of primary caregiving still chiefly rests upon informal family caregivers in Singapore, despite its developed eldercare infrastructure and services. This is due to the “underlying social policy framework, cultural values, and social expectations of filial duties in Singapore” [54] (p. 4).

Therefore, the sustainment of salubrious intergenerational relationships is necessary for caregiving, given that members of older generations become the primary responsibility of subsequent generations [55]. Intergenerational caregiving refers to caregiving activities that transpire between generations, which sometimes skip a generation [56]. Roles and responsibilities among family caregivers of older adults are often shared [57,58], although the intensity of care engagement differs across each family member [59].

Primary caregivers are perceived by themselves and others as the foremost source of assistance in most aspects of daily care for care recipients. They tend to be the primary point of contact for formal and informal networks [60]. Some are supported by other relatives or friends recognised as secondary caregivers, who execute tasks at a level akin to primary caregivers yet without the same level of responsibility, attributable to their comparatively less intensive and/or frequent assistance [61,62,63]. This includes hands-on help, personal care, assisting with instrumental ADLs, emotional support, health-related care [64,65], and respite care. This helps mitigate distress and burden among primary caregivers, thereby delaying and preventing the institutionalisation of care recipients [66]. 

However, scholars contend that secondary caregivers, typically younger with higher education and income, remain underexplored and overlooked in research and practice. Secondary caregivers experience comparable psychological distress and burden alongside physical strain, about whom researchers argue for inclusion in future research [62,67,68]. Yet, while there are avenues for information and services targeting primary caregivers, secondary caregivers face inadequate guidance on how best to support primary caregivers alongside themselves [61]. As caregiving roles develop with time amidst the vicissitudes of familial and labour market contexts (e.g., dual-income families, single-parent, or skipped-generation families), young individuals are also becoming an emerging population of primary caregivers [33,69,70].

### Research Gap and Present Study

Overall, Singaporean and Asian family caregiving studies fall short of understanding the experiences of young individuals who function as primary or secondary caregivers of older family members while traversing critical milestones of young adulthood. Given that addressing this knowledge gap amidst a rapidly ageing population is long overdue and merits prompt attention, we explored the lived experiences of caregiving for older family members by these young caregivers in Singapore. Specifically, this article addresses the following research questions:

(1) How do young caregivers in Singapore perceive their role in caring for older adults in their families?
(2) In what ways does caregiving pose challenges to young caregivers of older adults in Singapore?

## 2. Materials and Method

### 2.1. Conceptual Framework

Three conceptual lenses guided the present research. Firstly, the life course perspective is a valuable framework for researching intergenerational relations from developmental and historical perspectives, including individual and familial experiences that influence people at various life stages [71]. Pathway processes that connect earlier life trajectories and events with transitions into caregiving [72] remain relatively underexplored. Recent reviews question the adequacy of adopting a static view of determinants and contemporary time points of care transitions and caregiving effects. Hence, they call for capturing the complexities inherent within caregiving trajectories through the life course perspective, thereby taking account of the historical context of caregiver-care recipient dyads [73,74,75]. 

Secondly, while caregiving in Asian culture is commonly construed as collective familial responsibility, tensions and challenges are inevitable in the microcosm of family life. This necessitates exploring the dynamics within family caregiving experiences from an intergenerational perspective [56]. In this respect, intergenerational ambivalence aids with sense-making conflicting feelings inherent within the caregiving arrangement between the care recipient and caregiver [76,77]. 

Lastly, social exchange theory elucidates the costs (drawbacks) and rewards (benefits) encountered by young caregivers in their caregiving relationship [78]. Within Asian culture, intergenerational exchanges are emphasised throughout the lifecycle, where reciprocity through mutual assistance across generations is fundamental to maintaining intergenerational harmony [48], continuity, and cohesion, which define obligations and actions [79,80]. Considering such relational dynamics and predispositions would be integral to holistically understanding the caregiving process and journey of young caregivers.

### 2.2. Study Design

This study adopted the interpretative phenomenological analysis (IPA) methodology, underpinned by the fundamental principles of phenomenology, hermeneutics, and idiography [81]. These are consistent with the research aim of exploring the essence and intricacies of personal lived experiences of caregiving, along with meaning attribution and sense-making concerning such experiences [82,83].

### 2.3. Recruitment and Sampling Strategy

Participants were recruited through referrals from researchers’ own personal networks (i.e., friends and colleagues) using the word-of-mouth approach [84]. However, the individual participants referred to were not known to researchers personally prior to the study. This helped glean uninhibited and impartial opinions, which might otherwise be restrained by participants if recruited through formal gatekeepers (e.g., government-linked social service or non-profit agencies) from whom potential participants might have received direct services or assistance.

Prospective participants were screened to ascertain their suitability against the selection criteria informed by relevant research (see Table 1). Consistent with IPA’s methodological imperatives, this study achieved reasonable (demographic) homogeneity underpinned by purposive sampling [85,86]. To further maximise homogeneity, the final sample comprised only participants who were current caregivers (i.e., care recipients were still alive) and co-residing with care recipients, substantiated by extant research on the depth of caregiving involvement and burden experienced through such living arrangements [44,87]. This facilitates gleaning sufficient perspectives into the caregiving phenomenon amidst adequate contextualization [88]. 

This study recruited six co-resident young caregivers in their twenties, aged between 23 and 29, with an average age of 26 (see Table 2). The sample size is highly congruent with Smith’s et al. [81] (pp. 51, 52, 106) recommendations for conducting IPA research and methodological accentuation for small sample sizes. This helps facilitate an in-depth and meticulous analysis of each participant’s account amidst the complexity of the caregiving phenomenon [91], which will otherwise be hampered by large datasets. Such sample sizes are also consistent with prior caregiving-related IPA studies [92,93,94,95].

### 2.4. Data Collection

Semi-structured in-depth interviews were conducted over the Zoom video conferencing software per participants’ preference and convenience. Each interview lasted approximately 90 min and was audio-recorded and transcribed verbatim [96]. An interview guide constituting open and expansive questions was developed to facilitate a natural flow of conversation (Supplementary Material S1) and to minimise the influence of interviewer probes. Probes were only meant to encourage further discussion and were specific to participants’ responses. Hence, participants’ voices remained central throughout the interview and were devoted to uncovering their lived experiences.

Further, the photo-elicitation technique was incorporated by requesting participants, prior to interviews, capture images that meaningfully reflected their caregiving experiences to generate supplementary discussion. Leveraging photos enabled the unveiling and deepening of experiential significance by evoking memories, ideas, and emotions that may otherwise remain buried [97,98]. Crucially, this empowered the young caregivers to foreground their lived experiences on their own terms and frame of reference [99], heeding the calls of Hanson et al., [100] and Joseph et al., [15] to infuse participant-led and participatory approaches in the exploration of young caregivers’ experiences and as an ethical means to respect their agency and opinions. Photographs were not used as data for analysis per se but as stimuli to deepen discussions.

### 2.5. Data Analysis

Interview data were analysed using the systematic stepwise approach of IPA. First, each transcript was read and re-read for data immersion and familiarisation. Subsequently, comprehensive initial notes were documented on the right margin of the transcript, which were descriptive, linguistic, and conceptual in nature. Descriptive comments are centred on the content of narratives, such as noting important phrases, explanations, and summaries. Linguistic comments concerned language use, such as pronouns, repetitions, hesitations, and tonality denoting observed emotions. Conceptual comments entailed interpretative annotations of the narratives at an abstract conceptual level [81] (pp. 83–90).

These provisional notes were then re-examined against the associated datum before being transformed into several emergent themes for an increased level of conceptualisation. These themes were subsequently sorted and categorised into subordinate themes, which were, in turn, interrogated for possible connections and clustered into superordinate themes for the case. These exemplify a double hermeneutic process whereby participants foremost engage in sense-making and meaning-making of their lived experiences, which thereafter are decoded and interpreted by researchers [101].

In keeping with IPAs idiographic commitment to detailed and intensive analytical treatments of each case, this process was repeated for each transcript preceding the search for thematic patterns and integration across cases. This yielded insights into convergence and divergence within the sample through shared higher-order thematic qualities alongside unique idiosyncratic differences [102,103,104], which culminated in a master table of themes.

### 2.6. Reflexivity and Study Rigor

Prior to commencing training in gerontology and pursuing the current study, the first author worked as a researcher in the social service and non-profit sectors for nearly five years, during which dedicated research and awareness of young caregivers were non-evident, except in media features [10,11,105,106]. Further, the first author identifies as a young adult secondary caregiver for more than five years for his grandmother with dementia and comorbidities while supporting his primary caregiving mother. The researcher’s contemplation about the journey of caregiving in young adulthood served as an impetus to initiate this study, driven by the desire to learn from and deepen knowledge about the experiences of other young caregivers, who have been perceived as uncommon and “forgotten” [107]. This attitude is aligned with the lack of general societal awareness and local literature exploring caregiving experiences for older adults in earlier life stages. To this end, entering this research required the researcher to contend with potential presumptions regarding caregiving challenges. 

Owing to the phenomenological and hermeneutic inquiry of IPA, reflexivity is integral to recognising the researcher’s worldview and how participants’ experiences are interpreted [108,109]. Husserl’s work provided the premise to reflect upon preconceptions and lived experiences and minimise them through bracketing insofar as possible [110,111]. In respecting reflexivity, the researcher maintained an informal research diary and ensured an open-minded disposition throughout the research process to curtail suppositions. This included acknowledging that participants’ experiences may differ vastly from the researcher’s own.

Also, while the researcher shared caregiver status with the participants, he was not co-residing with his care recipient, unlike the study participants. Further, both authors belong to an ethnic group different from the participants. These helped to suspend assumptions and aid the phenomenological method [112]. Integrating the photo-elicitation technique further ensured that participants led interview discussions on their terms and frame of reference through their own photos, which further mitigated the potential presumptions of the researcher. Further, the immersive reading and intricate levels of noting of each transcript, guided by the stepwise analytical approach of IPA as described earlier, ensured the researcher stayed close to the participants’ narratives of their lived experiences. In particular, the iterative process between the etic (sense-making data through conceptual and theoretical lenses) and emic (reining in reductionism) perspectives as part of the hermeneutic and idiographic analytical cycle ensured the developing interpretations were grounded in the participants’ lifeworlds [91,96].

In adhering to the quality criteria of IPA, the (subordinate) themes considered for inclusion were based on a recurrence criterion of at least being present in half of the study sample (i.e., in at least three or more participant interviews) [104]. The developing themes were also discussed with the second author (who has over 25 years of experience as an academician, social worker, and gerontologist) for further refinement, condensation, and examination of connections between them. This collaborative work in IPA helped challenge the researchers’ inner-world preconceptions, assumptions, and interpretations to strengthen the coherence and plausibility of interpretations [113]. This reflexive dialogue, in turn, enhanced researchers’ reflexive engagement with the data and the study’s rigour [108].

Given that the first author who conducted the interviews is also a young adult, genuine rapport with participants was built with relative ease, enabling the promotion of egalitarian relationships and mitigating power imbalances between researcher and participants [114]. This enabled participants to express challenges with candour and without much hesitation or reservation, as evident in the tenor of the participants’ quotations, which otherwise may have elicited socially desirable responses if the interviewer was older. This contributes to strengthening the credibility of the findings.

Member-checking was executed by sharing interview transcripts with participants to ensure accuracy and anonymity, along with appending supplementary questions to seek further clarification and depth based on the issues discussed. These responses were incorporated as part of the interview data for analysis. An audit trail in the form of an informal research diary, transcripts, recordings, and analytical memos was maintained. Overall, rigour in the analysis and narration of findings was addressed by employing guidelines delineated by Smith and colleagues [81,102]. Collectively, these measures enhanced the study’s dependability, credibility, and confirmability [115].

### 2.7. Ethical Issues

Ethical approval was obtained from an ethics review committee appointed by the Singapore University of Social Sciences (approved 18 October 2022, approval reference code: 2022-GER 688IRB-001). Participation was voluntary, and the study’s aim and nature of involvement were communicated verbally and in writing through a letter of invitation and participant information sheet (PIS) during recruitment. Written informed consent was acquired before the commencement of interviews. Anonymity and confidentiality were ensured by assigning pseudonyms to participants and removing all potential identifiers from the data collected.

Given the emotional nature of the interviews, participants were followed up a few days after the interview to ascertain their well-being. This included providing a customised list of self-help resources based on concerns raised during interviews, in addition to the contact details of relevant SSAs and government schemes/assistance delineated within the PIS during recruitment. The interviewer also remained vigilant throughout the interview by empathetically listening and attending to, summarising, and reflecting back on participants’ responses, which are deemed “powerful cathartic facilitators” [114] (p. 1153). To this end, this study may have presented unexpected benefits, as most of the young caregiver participants perceived the interview as a rare opportunity to reflect deeply and openly about their caregiving experiences. These approaches helped ensure the ethics of care for the participants [114,116].

## 3. Results

This article presents six emergent superordinate themes alongside seven subordinate themes (see Table 3). Within illustrative participant quotes, capitalised words denote vocal accentuations by participants [117] (p. 539), [118], while square brackets ([]) indicate referents, clarifications, or omitted materials. Participants’ quotations may not be grammatically proper due to the colloquial use of English in Singapore [119,120]. To preserve the cultural essence, only minimal grammatical corrections were made, when necessary, without altering the semantics.

In essence, analysis unveiled young caregivers’ transitions into caregiving and the challenges encountered in their caregiving journey. The latter involves experiences of role conflicts amidst expectations and intergenerational conundrums. A brief description of typical photographs produced by young caregivers will precede the presentation of thematic findings hereafter.

### 3.1. Photographs Chosen

**Figure 1:**
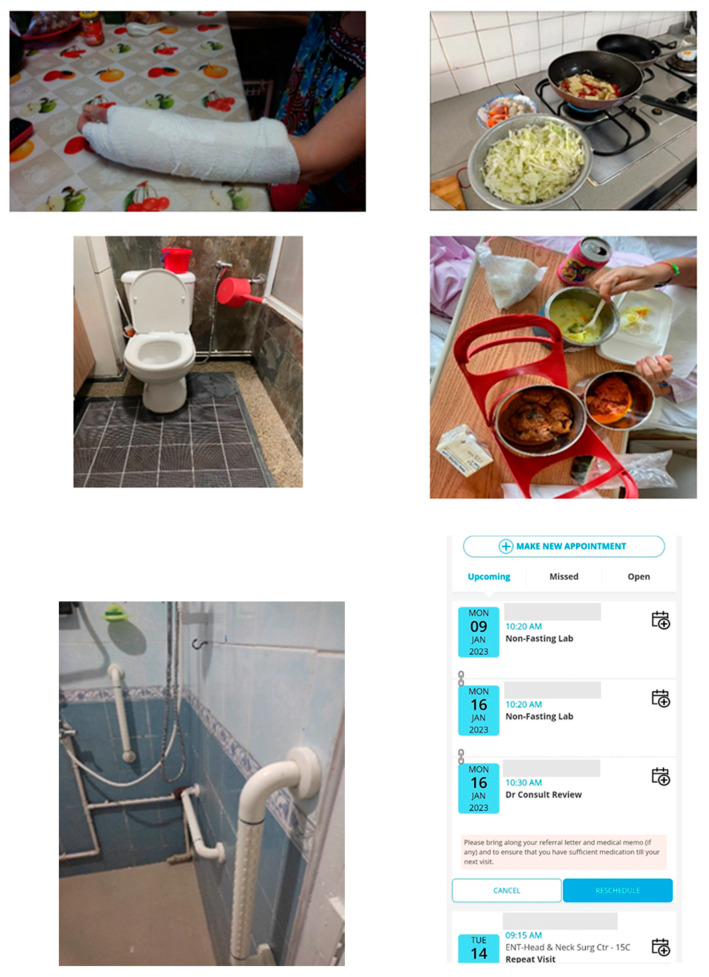
Examples of photographs presented by participants.

Most photographs shared by participants were of living spaces and objects, which symbolised their daily caregiving activities. Young caregivers used these pictures to narrate challenges encountered, such as nocturnal caregiving by assisting the care recipient to the restroom and with personal care, meal preparations and feeding, concerns over the safety of living spaces (e.g., hoarding by the care recipient), guilt over the care recipient’s fracture from a near-fall event, attending several medical appointments and medication administration, etc. (see Figure 1 for examples of participants’ photographs).

### 3.2. Transitions into Caregiving

This superordinate theme outlines the nature of young caregivers’ transition into caregiving as either by familial circumstance or by choice through self-selection and/or by other intra-familial members.

#### 3.2.1. Transition by Choice of Oneself and Others

Secondary young caregivers illustrated the pathway into caregiving through self-selection by themselves and others. This was underpinned by cultural imperatives, resulting in “*trying to help as much as I can in the household*” (Farhana). Imran’s transition to caregiving was a result of his co-residing mother’s decision to assume the role of primary caregiver from the extended family:

“*…what my mum [primary caregiver] thinks is that it doesn’t look good whereby a sister-in-law, non-blood related, to be taking care of him [care recipient]…So it’s like, why would you want someone else non-blood related to be taking care since he is your blood related…my mother told them never mind, she will take care of him since my mother was about to retire…*”

(Imran, 29, M)

Imran’s repeated mentions of “*non-blood related*” in a debatable manner (“*why would you want someone else*”) imply his concurrence with the premise of consanguineal kinship, on which his mother had chosen to assume caregiving. This transition informs Imran’s exposure to caregiving by having to assist the primary caregiver; “*she will definitely ask for my help*”.

Conversely, other participants reasoned their transition through self-initiation:

“*I just recently got married a year ago and moved to live with my in-laws…I live in a different environment with my husband’s uncle [care recipient] who has intellectual disability…you will have to volunteer yourself more because I’m a daughter-in-law. So, YOU as a daughter-in-law also have responsibility to help out mother-in-law [primary caregiver]…*”

(Farhana, 28, F)

Marriage as a life transition informed Farhana’s transition into caregiving. In addition, the term “*volunteer*”*,* alongside the affirmative use of “*YOU*” in relation to one’s position as a daughter-in-law, indicates a sense of familial obligation towards caregiving. This signifies internalised cultural and gender expectations around care in relation to the perceived socio-cultural organisation of the family unit.

Similarly, Samuel’s decision to pursue caregiving was based on a sense of duty towards his mother as the primary caregiver amidst the gradual observation of her caregiving distress: “*I have seen my mom very stressed out…That’s why I have to take care of grandma to reduce my mum’s burden.*”.

#### 3.2.2. Transition by Circumstance: “*I Was Put into This Situation*.”

The other half of participants, being primary young caregivers, transitioned from circumstances beyond their control and volition. Two of those participants were estranged from their parents, which led them to co-reside with grandparents who became care recipients with age:

“*So this [caregiving] is actually due to my family situation. My parents got divorced. None of my parents actually wanted to take responsibility of me and my older sister, so we ended up staying with my paternal grandparents…*”

(Xiahui, 25, F)

Xiahui’s mention of “*actually due to*” emphasizes her direct attribution of caregiving to familial circumstances, underpinned by a sense of being neglected by her parents. This was accentuated by her mentioning, “*…it [caregiving] doesn’t sit well with me because I was PUT into this situation.*”, perceiving her transition into caregiving as involuntary and unjustified while feeling trapped.

Similarly, Ryan ascribed his caregiving transition to troubled familial circumstances with abusive parents, which led to co-residing with his ageing grandmother:

“*…we suffered domestic abuse from our parents, so that led to us shifting out [to grandparents’ residence]…I would say the primary cause of me being a caregiver for anybody is that it just became the set of circumstances that led it to develop very organically…*”

(Ryan, 23, M)

Unlike Xiahui, Ryan alludes to having settled into the caregiving transition by characterizing it as an “*organic*” development over time. This was further evident through his rationalization of the transition as him being a “*first-born grandson who was actually capable of providing care, and it also factored in my unique skill set*”, as he hails from a nursing background. Although Xiahui and Ryan share a similar set of familial circumstances that led to caregiving, the latter’s account suggests upholding a sense of ownership towards caregiving through identifying with his familial and professional positions.

Conversely, beyond one’s control, Siti’s caregiving was due to her single mother’s relatively abrupt medical diagnosis:

“*my mom got diagnosed with a brain tumour last year…that was how I first got into caregiving…I just assumed that responsibility cause, I thought like it was supposed to be my duty anyway…because I’m the oldest, and I have to take care of my three younger siblings, basically being the breadwinner*”

(Siti, 25, F)

Like Ryan, Siti’s repetitive mention of “*I*” alongside expressing explicit concern for her siblings asserts her identity and familial position as the “*oldest*” child. These underscore her sense of familial obligation and ownership, informing her caregiving transition.

### 3.3. Grappling with Role Conflicts and Expectations: “I’m Still Trying to Find My Balance.”

Having addressed the context of young caregivers’ transition into caregiving, this superordinate theme shifts to explore caregiving’s intersection with other life domains. It examines young caregivers’ challenges with managing conflicting demands from other vital roles and responsibilities alongside caregiving, whose pressures were described as “*overwhelming*”, “*disruptive*”, and “*compromising*”. Young caregivers also battled with caregiving expectations arising from within and others.

#### 3.3.1. Amidst Competing Demands: A Conflicted and Constrained Life

All participants grappled with competing demands, primarily across work and/or school commitments, while caring. For young caregivers who were attending tertiary education and making ends meet through part-time work, caregiving compounded their existing responsibilities and resulted in making compromises:

“*I started weighing it out very logically; with the amount of schoolwork that I have now, and with the need to start earning some money to provide for the household, I do not reasonably have time for CCAs [school-based Co-Curricular Activities]… it is either I don’t starve, and I have money, and I’m working, or I have good grades, but I have lesser money, or I can forsake both of these and then have CCAs…there’s only so many hours in a week that I can work while not compromising my caregiving and school.*”

(Ryan, 23, M)

Ryan seems to be feeling strapped by financial constraints warranting the need to work while balancing one’s choices in the educational realm. This is evidenced by Ryan contrasting possible realities between “*I don’t starve*” by working, performing academically well, or pursuing CCAs. Other participants also grappled with financial constraints for caregiving due to financial inadequacies amidst early adulthood and career development: “*I’m just a fresh graduate. So, it’s very hard for me to contribute…I just paid off my student debt…there’s a lot of financial worry…I am giving grandma’s pocket money, pay for the Grab to Polyclinic…it adds up*” (Xiahui).

Conversely, caregiving intervened in the working lives of some employed young caregivers.

“*…when I’m working from home, and she [primary caregiver] has something to attend to outside, I would have to be available to him [care recipient]… sometimes it can get VERY OVERWHELMING [suppressed smile alongside a sigh]…But there’s no one else around…Sometimes he would bang on my door. So, when you’re in a meeting, it’s very hard, right? you have to excuse yourself…*”

(Farhana, 28, F)

Farhana seemed to confront pressing circumstances while working from home, where caregiving and work demands collided amidst the unavailability of the primary caregiver. Features of her struggle were built into Farhana’s tonality, and suppressed expressions were observed during the interview.

Working young caregivers also expressed concerns over the perceived intrusion of work etiquette, performance, and career progression amidst conflicting caregiving demands. This was infused with a sense of apprehension towards not living up to career-driven expectations from perceived others in the workplace:

“*when demands of caregiving are too high, it can affect my work, my performance you know, just suddenly taking urgent leaves…It’s my first year at [work organisation], and it’s my first career… My performance worse or something? They might appraise me? I don’t know. Because everyone else is not really taking that many [leaves]. In the first year, people expect you to grind, to always learn, and to have a lot of determination. Part of me feels that I’ll be left behind…*”

(Xiahui, 25, F)

Further, a sense of obligation towards caregiving and assisting the ageing primary caregiver also intercepted marital aspirations for independent living: “*Actually we [co-caregiving spouse] have intentions of renting out a flat while waiting for our house to be ready…but because of this [caregiving] situation, we made the decision to just stay and be there for her [primary caregiver].*” (Farhana).

Caregiving also affected young caregivers’ self-care and personal time amidst competing responsibilities. Some shared, “*I don’t have time for myself*” (Xiahui), and “*socializing with friends is restricted*” (Samuel). Others endured physical symptoms from caregiving strains that impacted daily functioning:

“*disrupted sleep and disrupted rest…over time I felt I was burning out. I was REALLY VERY tired, and I was starting to show symptoms like headaches…all kinds of back and body pain…I also had fever because I was caring…I felt I wasn’t really my best when I came to school or work…but I will always reduce it [physical symptoms] to, “It’s nothing. It’s nothing compared to my mom [care recipient].*”

(Siti, 25, F).

Siti’s various physical symptoms accentuate her experience of physical strains, though she underestimates them (repetition of “*it’s nothing*”) by comparing them to the perceived physiological stress of her mother. This suggests suppressing one’s caregiving strains despite their impact on other roles by embracing empathy and filial obligation.

#### 3.3.2. Perceived Inadequacy Amidst Role Identity and Expectations

Four participants reflected on their caregiving role by perceiving one’s own expectations in relation to their familial position. Some participants expressed perceived inadequacy in their caregiving role and falling short of one’s filial duty: “*…just feeling I’m not good enough daughter… I wasn’t doing enough for her. I had a lot of self-doubts… I had these expectations on myself that I I should be doing MORE…*” (Siti). The repetitive mentions of “*I*” assert the participant attached a strong filial identity to her caregiving role, suggesting value-laden expectations ascribed to caregiving that remained unsatiated.

Conversely, some participants’ perceptions of their caregiving role were negatively influenced by others within the family. Farhana’s perceived inadequacy in both her assistive roles as secondary caregiver and daughter-in-law is informed by the critical demeanour of the primary caregiver: “*Her [primary caregiver] personality, she’s quite critical…So it’s very hard to be ENOUGH, to be a good enough daughter-in-law…*”. Distinctively, Xiahui derives perceived inadequacy from her caregiving relationship with her grandmother:

“*It’s a very ONE-SIDED kind of relationship…ALWAYS ME like helping her [care recipient], but I understand she’s old. Yeah, like there’s NO VALUE for me…It is always about her needs and wants. Besides forcing myself to think feel good because her needs and wants are fulfilled, what is in it for me?*”

(Xiahui, 25, F)

Xiahui seems to grapple with the normative expectations and nature of helping an older individual. The mention of “*NO VALUE for me*” implies feeling unappreciated by the care recipient in her role as a caregiver. This seems to stem from her perceived discrepancy in relational expectation due to the perpetual nature (“*ALWAYS me helping*”) of unidirectionality and lack of reciprocity (“*ONE-SIDED*”) within the care relationship. This leads her to the existential question, “*What is in it for me?*”.

### 3.4. Navigating Intergenerational Dynamics and Relational Distress

Having examined young caregivers’ role identity and expectations, this superordinate theme extends to explore relational complexities faced within the intergenerational context of caregiving, encompassing middle-generation adults and care recipients.

#### 3.4.1. Resentment over a Lack of Support from the Middle Generation

Participants expressed disappointment and frustration over the lack of involvement and genuine engagement in caregiving by their middle generation and extended family. One participant expressed resentment over the abdication of caregiving responsibility:

“*My grandma has FOUR children, but none of them is really taking responsibility, and one of them is my dad, who is not only not taking responsibility for his CHILDREN, but his mum as well…to put it bluntly, it PISSES ME OFF!… [participant’s frustration visible through facial expression].*”

(Xiahui, 25, F)

Xiahui’s use of expletives reflects a palpable depth of frustration and aggrievement over the middle generation’s lack of caregiving responsibility. This is compounded by her father, who is alluded to as having abandoned his familial role and responsibility towards both generations.

Paradoxically, secondary young caregivers grappled with imposed expectations from non-caregiving extended families: “*It’s quite difficult to balance… and manage people’s [extended family] expectations… I mean they are not caring, why are they talking SO MUCH!?*” (Farhana). The lack of empathy and understanding from such relatives was attributed, with assertion, to caregiving realities being out of sight to them: “*They [aunts and cousins] don’t see me and my mum’s [primary caregiver] difficulty. They say,* “*she’s [care recipient] fine leh not like acting out*”*. But WE are the ones that live together with her. WE ARE THE ONES first-hand experience…*” (Samuel).

Secondary young caregivers empathized with their distressed adult primary caregivers amidst expectations and conflicts from extended families, despite experiencing secondary distress:

“*when I see her [primary caregiver] unhappy with those words that they [extended family] say… over critical about the way she cares for her brother…it is also very unsettling for us. We don’t want her to be stressed out…Sometimes you know stress can affect the people around you…she would be more critical.*”

(Farhana, 28, F)

Relational conflicts also arose from the middle-generation extended family’s lack of trust and differences in viewpoints, which infuriated participants like Imran:

“*They [middle generation extended family] don’t trust us in taking care of our uncle [care recipient]. But I have been taking care of my uncle since back then … this kind of family issues are SOO STUP..I I’m sorry…It’s SO NONSENSE to hear and see this family feud. It is VERY HEARTBREAKING to get it from your own aunty and uncle…if older people talk to you, you will always lose lah…because you need to respect them, you can’t voice out yourself…they think what they say is best for him [care recipient]*”

(Imran, 29, M)

Frustration with extended family members is evident, with Imran holding back his language. Emphasising family feuds as “*HEARTBREAKING*” suggests deep disappointments from relational strains with relatives on whom Imran may have had affectionate expectations. Imran also expresses a sense of resignation from the power dynamics inherent within intergenerational differences in viewpoints regarding caregiving amidst cultural expectations to exercise deference.

#### 3.4.2. Sense of Agency in Shouldering Care

Amidst familial tensions and a lack of support, participants alluded to “*taking on more of the caregiving role*” (Ryan) through exerting agency. Siti recounted the critical period prior to her mother’s surgery, where she acted decisively in medical decision-making amidst being conflicted by middle-generation extended family, “*crazy because I was directly dealing with life or death situation for my mum, and overwhelming because of conflict of opinions from my [extended] family members…I just felt I should step up and just go on with the surgery.*” As the eldest sibling, Siti also assumed her mother’s role in providing for her younger siblings.

Relational conflicts with extended family also resulted in participants deciding to shoulder care responsibilities. Imran exercised a protective role for his primary caregiving mother amidst conflicts and assumed financial responsibility in caregiving to prevent the recurrence of family feuds regarding financial matters:

“*I always told her [primary caregiver] this. If they [extended family] ask, tell them that, “My SON has money. So I can ask from my son”. I wasn’t happy with my mother’s side…I fought for my mother. I fought for my mother…I didn’t want her to go through all this SHIT again and MESS again…*”

(Imran, 29, M)

The repetitive mentions of fighting “*for my mother*” reflect the young caregiver’s agency based on an underlying sense of affection and attachment towards the primary caregiver. This also resonated with other secondary young caregivers, which serves as a motivator for them to assume a greater role in caregiving. It further signifies ambivalence amidst the coexistence of solidarity and conflicts in intergenerational ties amidst caregiving.

#### 3.4.3. Distress from Care Recipient’s Behavioural Challenges

In addition to relational complexities with the middle generation, five participants shared feeling troubled by care recipients’ challenging demeanour. This led to some participants confronting the nature of caregiving itself: “*When she [care recipient] says mean things, that’s where I get very, very frustrated, I get very hurt. And that’s when I just really HATE caregiving.*” (Xiahui). For Samuel, his distressed self-questioning on the need for caregiving is palpable, from perceiving the care recipient’s favouritism:

“*So the biasness…she treated the two cousins WAY better than me…in EVERY aspect! I kept questioning myself…why, why, why am I, why am I taking care of her? Why is it me? Why not just call the two cousins to take care of her instead? It will be much more easier. She’ll probably have a better, HAPPIER LIFE LAH!…I think deeply, but the thing is that…I still can’t get a very definite answer…*”

(Samuel, 26, M)

The repetitions of “*why*” denote existential questioning, underscoring the struggles of finding one’s purpose in caregiving amidst relational dissatisfaction. Amongst other participants, “*stress is escalated when making medical decisions*” (Ryan) amidst the lack of care recipient’s willingness to cooperate. Others relayed a sense of fear (“*you’re AFRAID that he [care recipient] will lash out even more*” (Farhana)) and emotional outbursts (“*last time, I couldn’t hold my temper…he [care recipient] will SHOUT at you back…*” (Imran)).

Despite feeling distressed, participants engaged in problematising and rationalizing care recipients’ personhood, implying an effort to reflect upon their care recipients’ challenging behaviours, which also signals a sense of ambivalence. Samuel perceives his care recipient “*doesn’t really understand because her mental health state is like in…young children that kind..*” whilst Imran similarly likens his uncle’s state of being to a young person, which influences his caregiving interaction:

“*We see him [care recipient] as an older person. But he sees himself as a younger child… If he’s mad or anything, just treat him like because he’s in the mind of a five-year-old. So, you need to treat him like a five-year-old person.*”

(Imran, 29, M)

In contrast, Farhana dissociated the care recipient from his intellectual disability, evident in the language of her explication: “*I do feel sad for him that he came into that circumstance…his brain, he would overthink, and then he will lash out…I know it aggravates others, but he can’t do anything about it*”.

Given the inherent relational challenges, some participants acknowledged the risks arising from caregiving distress for both caregivers and care recipients, articulated as externalised general observations. Such risks that the participants were cognizant of included depression, suicidality, and homicidality. For instance, Imran articulated, “*…nowadays people’s minds they can run wild. If they fall down deep, they WILL definitely fall down deep. They will start thinking about suicide. You can go into depression and ALL sorts of things.. it would affect you [caregiver], but then it will also affect the person [care recipient]*”. The repetitive mention of “*fall(ing) down deep*” into one’s thoughts that may “*run wild*” amidst stressors underscores Imran’s understanding and perceived potency of rumination as antecedents of suicidality and depression. Notably, Farhana conveyed the risk of homicidality from caregiver burden by citing an example of an infamous culpable homicide by a father killing his twin sons with autism spectrum disorder in Singapore, which was reported in the media [121]:

“*I think it’s quite a challenge. You know you have heard of cases where they do bad things to their kids with intellectual disabilities [autism]. So, it CAN get to that point, I understand… I mean, you know about the father who killed his twins who had autism?… I think they found the kids at the reservoir [canal] or something… So, you know it gets you when you hear stories like this, you get very, sometimes it can get very overwhelming…because if you don’t cope with it, it’s stressful for the person who is providing care [and it] is also stressful for the person who is with the disability, or you know the person who needs care. ‘cause they [care recipient] can’t, sometimes THEY CAN’T communicate how or what they want. So, when someone who is caring for them is going through stress, it can be dangerous even for THEM [care recipient]*”

(Farhana, 28, F).

While characterizing homicidal behaviour as sinful (“*bad things*”), Farhana’s account signals an empathetic understanding by illustrating it as a dreadful yet plausible consequence of the extremity of caregiver distress if left unmanaged (“*if you don’t cope with it*”). Such media reports seem to have induced a sense of apprehension and accentuated the perceived gravity of caregiver burden evident in the undertone of her narration (“*it gets you when you hear stories like this…it can get very overwhelming*”). This could potentially be attributed to her resonance with the current caregiving reality for her older family member with an intellectual disability, which could have informed her empathetic response of emphasizing communication challenges as being inherent for such a caregiver-care recipient dyad (“*they can’t, THEY CAN’T communicate*”).

## 4. Discussion

This study sought to explore the lived experiences and multifaceted challenges encountered by young caregivers of older family members in Singapore. In doing so, findings unveiled young caregivers’ transition into caregiving, experiences of role conflicts and expectations, intergenerational conflicts, and ambivalence.

### 4.1. Transitions into Caregiving

The findings uncovered the contexts predisposing young caregivers’ transitions into caregiving. Consistent with Day [20], young caregivers assumed primary or secondary caregiving roles based on an underlying sense of filial obligation and duty and/or the lack of alternatives as informed by familial circumstances. Hence, their pathway to caregiving may not solely be demarcated by care recipients’ ageing, illness, or disability but by earlier socially situated processes characterized by notions of diminished choice or non-voluntarism [20], as in the case of the primary young caregivers.

For some, it was informed by the lack of support and accountability of the middle generation from past life events of parental separation and neglect, leading to the formation of skipped-generation families. Leu et al. [122] corroborate that these young individuals assume greater responsibility within the family as they become older while co-residing with their ageing care recipients, with some not realizing how shouldering significant caregiving responsibility and filling in the gaps of absent adults were placing them in a new position within the family structure. Despite these ordeals, most young caregivers expressed a sense of agency in their transition, as corroborated by Blake-Holmes [123].

In explicating caregiving transitions as shaped by past life trajectories and events, this study has shifted away from the widely researched snapshot understanding of caregiving through individual-level predictors towards qualitatively exploring the pathways into caregiving [72]. Hence, this has contributed to addressing the literature gap on exploring the transition into caregiving roles from a life-course perspective [73,75,124], especially concerning young caregivers [125].

### 4.2. Role Conflicts and Expectations

Further, while existing research has explored adult caregivers’ challenges of balancing multiple roles in their lives [126,127], this study highlighted the conundrums of caregiving amidst the developmental tasks typical of young adulthood (i.e., tertiary education, career progression, and marital plans). Consistent with role theory [128], young caregivers experienced role conflicts and strains leading to exhaustion, tensions, and somatization [127,129]. Notably, past studies reported a dose-response relationship between the extent of care responsibilities and somatic symptom burden, alongside mental health, insomnia, and impaired sleep quality, including cortisol dysregulation, reflecting negative health outcomes among young caregivers [130,131,132,133]. Yet, of the embracing, compromising, and integrating strategies uncovered by Pope et al. [134] as utilized by young caregivers for caregiving, the present study’s participants mainly were seen to embrace their caregiving role with disruptions to daily life. Others were making compromises to sustain caregiving roles.

For young caregivers who experienced disruptions while working, questions remain on how such circumstances might influence career development opportunities. Past studies indicate that caregiving by young adults interferes with employment, such as by missing out on job promotions and inadequate support at work [135,136]. Institutional pressures from work demands may compete for young caregivers’ time and resources, reflecting the notion of structural ambivalence, which could induce stress [137]. Scholars assert that compared to middle-aged and older caregivers, young caregivers are more likely to work while caring for others, thereby needing workplace accommodations (e.g., leaving early, taking time off), and are more likely to face greater financial strains [89,138]. Additionally, recent comparative studies involving young caregivers and non-carers found the former less likely to obtain a university degree and enter employment and had lower incomes from paid employment, with such outcomes negatively associated with caregiving intensity [27,139]. Hence, hindrances faced whilst pursuing higher education and career advancement are cautioned by researchers to place young caregivers at risk for accumulated social disadvantages over the life course [134].

Further, findings underscored the confluence of caregiving as an identity-relevant behaviour with perceived familial roles (e.g., daughter, daughter-in-law, grandson) to which meanings and expectations are attached [140,141]. For instance, filial piety is generally lauded as an Asian cultural underpinning for preserving familial relationships, though it presents mixed associations with caregiving outcomes [40,142]. Contrary to Lai [143], who found filial piety among Asian family caregivers to mitigate adverse effects of caregiving stressors, young caregivers from the present study seem to experience added pressure and perceived inadequacy amidst filial identity associations with their caregiving role. Despite some feeling troubled, abandoned, and stressed in their caregiving role, they felt obligated to care while compromising aspects of young adulthood and their well-being.

However, while most participants repeatedly associated caregiving as an extension of their familial role, the mention of being a granddaughter was much less evident in Xiahui’s transcript. Following Eifert’s et al. [140] argument, embracing the role and identity of caregiving is contingent upon the caregiver’s wilful choice to become a caregiver and the care recipient’s appreciation of the assistance rendered. The absence of these seems to negatively influence Xiahui’s perceived caregiving role and identity.

Consistent with the exchange theory, although caregivers may not possess expectations for material rewards, the emotional receipt of gratitude and love in exchange is anticipated, whose absence challenges the principle of reciprocity norm, as experienced by Xiahui [144]. Overall, the identity of young caregivers needs to be understood as being negotiated in the context of their familial social role and expectations, alongside the broader context influencing their transition into caregiving.

### 4.3. Intergenerational Dynamics and Relational Distress

Lastly, consistent with Lien & Huang’s [56] (p. 83) study on intergenerational caregiving challenges, intergenerational disharmony was perceived by young caregivers to ensue from “differentially adhered to traditional or contemporary values”, roles, and responsibilities. In fact, a lack thereof amongst the middle generation contributed to provoking most young caregivers, inducing intergenerational conflict amidst caregiving challenges, thereby complicating intergenerational relations [145]. This further highlights the socio-cultural and structural context within which caregiving complexity is situated, which is peculiar to Asian intergenerational families.

In some Western countries, such as the Netherlands, aged care provision becomes a state responsibility. Adult child caregivers indicate ambivalence about maintaining social contact rather than caregiving, reflecting their caregiving decisions being centred on visiting ageing parents rather than delivering care [146]. In Asia and Singapore, familial obligations and filial piety among adult child caregivers are deemed predominant reasons for caregiving to parents/parents-in-law, which influence the assumption of caregiving roles [147,148]. However, this study challenges such generalisations from past Asian research, as familial obligations were observed to be inconsistent across generations and absent among the middle generation of some young caregivers. This led to the younger generation shouldering the care of their older family members in some cases. In other cases, there was a filial concern for grandparents.

Young caregivers also expressed being troubled by care recipients’ challenging demeanour. While conflict is inherent in all close relationships to some extent, frequent conflict in a highly interdependent relationship, such as caregiving, can dominate the relationship to the exclusion of all else [149]. Van Bruggen et al. [150] corroborate that most challenges arise from the social-relational domain with the care recipient, who tends to be unaware of such a burden, as experienced by young caregivers. Nevertheless, Lyons et al. [149] argue for the need to glean perspectives from care recipients to uncover relational discrepancies and dynamism.

Further, as some young caregivers alluded to perceiving and treating their care recipient as a child, this suggests potential parentification of the youth [151]. However, it remains unclear whether the young caregivers in this study can be portrayed as parentified or simply caregivers. Nevertheless, providing training and skill development from a third party, modelling its approach, and what it looks like from the caregiver’s perspective may be beneficial to eliminate potential parentification in young caregivers [152].

While some of the participants were aware and concerned about the risks of caregiver distress in the form of depression, suicidality, and homicidality, the existence or extent of such ideation within participants were not uncovered, and neither were they explored in this study. Nevertheless, it is noteworthy that these have been identified as being of particular concern in adult caregiver literature, whereby caregiver burden and distress have been shown to contribute towards suicidality, elder abuse, and homicidality [153,154,155]. The latter warrants further exploration, given the current dearth of research in the local context on homicidal ideation among caregivers across age groups. O’Dwyer et al. [155] forewarn that it would not be unreasonable to construe that, under the right circumstances, passive thoughts or violence by caregivers may escalate to homicidal ideation or acts. This is pertinent given that a cross-sectional and non-randomised Singaporean study reported the prevalence of elder mistreatment in the community to be 8.3% and that a conflictual and hostile family environment, characterized by psychological and physical abuse, underpins and exacerbates mistreatment [156]. Yan’s et al.’s [153] typographic analysis of caregiver profiles in relation to elder mistreatment further found that isolated and traumatized caregivers reported higher levels of caregiver burden and stress, which were one of the greater risk factors for mistreatment, among other factors such as childhood traumatic experiences. Nevertheless, Burnight & Mosqueda [157] (p. 8) caution that while stress and caregiver burden play an indispensable role as contributing risk factors for elder abuse, these should be considered “without excusing the abuser’s behavior”.

On the contrary, caregivers have also been studied to be at risk of verbal and physical abuse by their older care recipients for whom they provide care [158,159]. This may potentially be congruent with Xiahui’s experience of being emotionally hurt by her care recipient’s remarks and demeanour, although whether this amounts to emotional abuse inflicted by the care recipient and/or arising from intergenerational communication challenges remains uncertain.

These studies, in essence, reflect the complex web of contributory risk factors at present and across caregivers’ and care recipients’ life courses, which could inform caregivers’ distress and burden. It is also well established that the human brain’s prefrontal cortex undergoes a “rewiring” process and development during adolescence that is not complete and attains maturation only until approximately 25 years of age [160]. Hence, young caregivers may naturally face challenges in processing relational and emotional distress, ambivalence, and complexities from caregiving, thereby necessitating targeted support.

Nonetheless, in the struggles and challenges of caregiving, young caregivers, as with adult caregivers, could also be employing coping strategies and experiencing personal growth such as enhanced maturity [161] (Fives et al., 2013), empathy, compassion, and resilience [162,163,164]. Netto et al. [165] further contend that the examination of caregiving experiences in extant literature has predominantly focused on caregiver burden, as most research has adopted a caregiver-centered, one-directional model whereby care recipients are construed as the source of burden and stress on the caregiver. Instead, Netto et al. [165] advocate for a caregiver-centered bidirectional model that embraces a holistic view, considering both the positive and negative influences of the uplifts and burdens among caregivers. Hence, while it is important to understand the detrimental aspects of caregiving, it is also equally critical to recognise the positive aspects and potential for psychological growth for young caregivers [15]. However, this was beyond the scope of this article, which addresses the current dearth of research on caregiving challenges among young Asian caregivers and is hence aimed at being explored in a separate publication by the authors.

In sum, this study has addressed the call to explore the emotional impact of being a young caregiver amidst the risk of stress and/or depression [166].

### 4.4. Strengths and Limitations

Generalisability is not the purpose of qualitative inquiry. Instead, the transferability underpinning qualitative explorations was enabled by knowledge development through the systematic and reflective process of inquiry [167]. This study has addressed the existing knowledge gap in the empirical and applied family caregiving literature in Singapore and the Asian context for young caregivers of older family members. Particularly, the use of IPA enabled in-depth exploration and understanding of the lived experiences and contextual meanings ascribed to the caregiving transition, associated intergenerational dynamics, and inherent challenges. This helped bridge literature gaps by uncovering the experiential significance of caregiving as embedded in intergenerational, life stage, and life course perspectives among young caregivers.

However, the small sample size poses limitations on the degree of transferability to other young caregivers. Nevertheless, as participants shared similar experiences with intensity and conviction evident through the themes, it substantiates the influential nature of caregiving for these young caregivers, which is suggestive of wider applicability. Readers could think in terms of theoretical generalisability by considering the findings based on their professional experience and knowledge when assessing the phenomenon’s extension [81] to other young caregivers.

### 4.5. Implications and Future Research

Findings from this exploratory study present implications across policy, practice (Supplementary Material S2), and research. Given that caregiving is interwoven with young caregivers’ life-stage concerns (e.g., education, early career, marriage) alongside intergenerational predicaments, greater partnerships between practitioners from family, youth, and eldercare SSAs are warranted to address associated challenges holistically. This necessitates adopting family-centered joint needs assessments.

Caregiver identification, assessment, and intervention should transcend the adult caregiver-care recipient dyad by considering other younger caregivers in the household to extend adequate support to this hidden population within family caregiving networks (see the Department of Health, UK’s [168] (2015) whole-family approaches to assessing caregivers). This could also help identify and enable potential secondary caregivers who could provide respite to and alleviate caregiver burden among primary caregivers [169]. Nevertheless, secondary young caregivers in this study played dual roles of caregiving to care recipients while supporting their adult primary caregivers amidst stressors, from which emotional strains compounded. Hence, these caregivers should not be ignored by an exclusive focus on primary and adult caregivers. Therefore, services need to respond flexibly to the family system, meeting the needs of both older care recipients and adult and young caregivers. Essentially, in assessing caregiver burden and risks, the primary, secondary, and tertiary caregiver systems should be considered. Otherwise, it results in the continuation of evidential bias that ultimately informs caregiving interventions and policy [170].

Further, as most young caregivers in this study were working and grappling with competing demands, employers and HR practitioners could extend flexible work arrangements. Progressive practices such as in-house counselling, support groups, and referrals to caregiver-related resources could help create empathetic workplaces. Young caregivers’ role conflicts within the educational realm, with some juggling part-time employment to make ends meet, also underscore the pivotal role of educational institutions in identifying and supporting young caregivers.

Policy levers and legislative frameworks are equally crucial to institutionalising support for young caregivers, thereby effectuating targeted service developments. Existing caregiving policies, such as Singapore’s home caregiving grant, whose stringent qualifying criteria could be made more accessible [171]. These are especially pertinent for young caregivers whose care recipients’ needs are below the eligibility threshold of three ADLs [172], yet they require support in sustaining caregiving while finding their footing early in their career and financial adequacy.

Future research should seek to establish the prevalence of young (primary and secondary) caregivers in Singapore and ensure the inclusion of minors aged below 21, which was beyond the scope of this study and of vast local caregiving research. Attention should be given to the type of care recipients’ illness (e.g., dementia) and its impact on young caregivers’ perception and experience of caregiving [33]. Given the current absence of national studies examining the state of young caregivers in Singapore, the use of validated instruments for this population as part of future representative research is imperative. Psychometric instruments developed in the UK for young caregivers, such as the Young Carers Perceived Stress Scale (YCPSS) and Positive and Negative Outcomes of Caring (PANOC-YC20) [173,174], could be validated for cultural transferability and adapted in the local context for administration to produce generalizable findings on the extent and nature of the effects of caregiving among young caregivers. These should also be examined longitudinally alongside the impact on developmental (e.g., education, career, courtship, marriage, and parenthood) and well-being outcomes to inform targeted interventions and support, as most participants expressed emotional stress from caregiving. This is critical amidst rising concerns over youth mental health and suicides in Singapore [175,176], which also demands an exploration into young caregivers’ coping, adaptation, and resilience. For instance, past research indicates that benefit-finding, as a form of cognitive reappraisal coping strategy through meaning-making, is associated with psychological adjustment, acceptance, problem-solving, resilience, and seeking social support [163,177,178], which could be leveraged by practitioners when working with young caregivers.

This study also highlights that young caregivers’ experiences were not contingent only upon caregiving demands or available resources but primarily also the existing family dynamics, amidst broader socio-cultural norms and associated expectations of intergenerational relations. Thus, future studies should further explore intergenerational caregiving experiences from a family systems perspective, including and integrating the voices of older care recipients, middle-generation caregivers, and young caregivers. This is especially pertinent amid the ageing or declining health of care recipients, whose effects would reverberate across the family system, potentially inducing greater responsibility among young caregivers [179].

Such future endeavours would enrich the extant young caregiver literature, which is predominantly from the west. This would pave the way for cross-cultural and cross-country comparative studies and permit novel assessments of how institutional and structural factors alongside cultural norms impact young caregivers [13].

## 5. Conclusions

To the best of the researchers’ knowledge, this is one of the first exploratory studies extending the current understanding of young caregivers in the literature against the socio-cultural context of Singapore. Young caregivers confronted challenges across role conflicts and expectations amidst developmental tasks and transitions while caregiving. This was compounded by experiences of intergenerational conflicts and ambivalence vis-à-vis embracing cultural tenets of filial piety and familial obligations. Most participants also experienced emotional stress. A word of caution is that if burnout among young family caregivers reaches a high point, elder abuse or even suicidal and/or homicidal thoughts may transpire.

Fundamentally, greater collaborations among multiple stakeholders interfacing with youths and seniors underpinned by family-centred approaches to assessments and service delivery are necessary. Findings also warrant further research to establish the prevalence and measure pertinent caregiving-related, developmental, and well-being outcomes of young caregivers. A whole-of-society approach is critical to enabling young caregivers to realise their full potential while contributing to their ageing families and nation.

## Figures and Tables

**Table 1:** Selection Criteria.

Inclusion Criteria	Exclusion Criteria
Young adults who: (a) were aged between 21 and 35 within the life stage of youth as defined by Singapore’s Ministry of Culture, Community, and Youth [31] (b) were an informal and unpaid caregiver to an older family care recipient aged 50 years or older [89] requiring support due to chronic illness, frailty, disability, or mental health conditions [44] (c) provided either physical, socio-emotional, or other instrumental care (e.g., attending medical appointments, participation in decision-making, supervising a foreign-domestic worker) at least once a week, and (d) provided at least 6 months of unpaid care preceding data collection [29,64]	Young adults: (a) whose care recipients have deceased more than 6 months before data collection [64] (b) whose care provision is only through financial support, and (c) not visiting care recipients once a week, minimally [90]

**Table 2:** Participants’ demographics.

Participant Pseudonym	Gender	Ethnicity	Age of Young Caregiver	Marital Status	Highest Education Level	Occupation	Co-Residing Family Members	Age of Cr *	Relationship of Cr	Primary/Secondary Caregiver
Samuel	Male	Chinese	26	Single	Diploma	University student	Parents and grandma	70	Maternal grandmother	Secondary
Farhana	Female	Malay	28	Married	Bachelor’s degree	Full-time employee	Spouse, mother-in-law, and uncle-in-law	64	Uncle-in-law	Secondary
Imran	Male	Malay	29	Married	Nitec ^	Full-time employee	Spouse, mother, and uncle	64	Maternal uncle	Secondary
Siti	Female	Malay	25	Single	Bachelor’s degree	Full-time employee (Fresh graduate)	Mother, and 3 younger siblings	50	Mother	Primary
Ryan	Male	Chinese	23	Single	GCE O-level **	Polytechnic student	2 younger siblings, grandma	68	Paternal grandmother	Primary
Xiahui	Female	Chinese	25	Single	Bachelor’s degree	Full-time employee	Grandma	77	Paternal grandmother	Primary

Note: * Care recipient; ^ National Institute of Technical Education Certification; ** Singapore-Cambridge General Certificate of Education Ordinary Level; Demographic homogeneity was achieved through young caregivers (a) being in their twenties, (b) being current caregivers, and (c) co-residing with care recipients. All care recipients only needed minimal-to-moderate assistance with activities of daily living. None are severely disabled.

**Table 3:** Recurrence of themes across participants.

Superordinate Themes	Subordinate Themes	Samuel	Farhana	Imran	Siti	Ryan	Xiahui
1. Transitions into Caregiving	1.1 Transition by choice of oneself and others	Yes	Yes	Yes	-	-	-
	1.2 Transition by circumstance: “*I was put into this situation.*”	-	-	-	Yes	Yes	Yes
2. Grappling with role conflicts and expectations: “*I’m still trying to find my balance.*”	2.1 Amidst competing demands: a conflicted and constrained life	Yes	Yes	Yes	Yes	Yes	Yes
	2.2 Perceived inadequacy amidst role identity and expectations	Yes	Yes	-	Yes	-	Yes
3. Navigating intergenerational dynamics and relational distress	3.1 Resentment over lack of support from the middle generation	Yes	Yes	Yes	-	Yes	Yes
	3.2 Sense of agency in shouldering care	Yes	-	Yes	Yes	Yes	-
	3.3 Distress from care recipient’s behavioural challenges	Yes	Yes	Yes	-	Yes	Yes

## Data Availability

The full interview data used in the research cannot be publicly shared because of ethical clearance obtained on the basis that data are retained by the study’s research team alone for confidentiality, except for participants’ quotations and pictures. The Supplementary Materials include the interview guide used in the research and all the data relevant to the themes presented in the Results section.

## Supplementary Materials

The following supporting information can be downloaded at: https://www.mdpi.com/1660-4601/21/2/182/s1, File S1: Interview Guide; File S2: Recommendations for Policy and Practice; File S3: Themes and Additional Participant Quotations. Reference [180] is cited in the Supplementary Materials.
