# Supplementary material for: Exploring the Lived Experiences of Caregiving for Older Family Members by Young Caregivers in Singapore: Transition, Trials, and Tribulations
Source: Int J Environ Res Public Health. 2024 Feb 5;21(2):182. doi: 10.3390/ijerph21020182 (PMC10888348; doi:10.3390/ijerph21020182)
Supplement: Supplementary file 1 [file ijerph-21-00182-s001.zip › File S1 - Interview Guide.pdf]

Interview Guide

Thank you for agreeing to participate in this research on caregiving for older family members by young caregivers.

Key Anchor Questions Addressed During Interviews:

1) Please tell me about yourself and what you are currently doing.

2) Could you describe how you came about providing care for your older family member?

3) How would you describe your role in providing care for your older family member?

4) How would you describe the experience of providing care for your older family member? Please tell me about the photo(s) you had taken and why?

5) What are some of the challenges or concerns you have about providing care?

6) In what ways has providing care and support interfered with or impacted your life?

7) On a scale of 1 (not at all stressful) to 5 (very stressful), how emotionally stressful is it for you to be caring? Please, could you explain your rating?

1___________2_____________3___________4___________5

Not at all stressful   Very stressful

8) How do you cope with these challenges?

9) How have you gained or benefitted from caring for your older family member?

10) What does providing care to your older family member mean to you?
