# Supplementary material for: Exploring the Lived Experiences of Caregiving for Older Family Members by Young Caregivers in Singapore: Transition, Trials, and Tribulations
Source: Int J Environ Res Public Health. 2024 Feb 5;21(2):182. doi: 10.3390/ijerph21020182 (PMC10888348; doi:10.3390/ijerph21020182)
Supplement: Supplementary file 1 [file ijerph-21-00182-s001.zip › File S3 - Themes and Additional Participant Quotations.pdf]

Supplementary Material 3: Themes and Additional Participant Quotations

| Superordinate Themes | Subordinate Themes | Additional Illustrative Quotes |
|---|---|---|
| 1. Transitions into Caregiving | 1.1 Transition by choice of oneself and others | "...what my mum [primary caregiver] thinks is that it doesn't look good on them whereby a sister-in-law, non-blood related to be taking care of him [care recipient]... So it's like why would you want someone else non-blood related to be taking care since he is your blood related…So my mother told them [extended family], she will take care of him since my mother is about to retire at that time." (Imran)<br><br>"I have seen my mom [primary caregiver] very stressed out. Grandma [care recipient] may give hard time to my mother...That's why I have to take care of grandma to reduce my mum's burden..Because I have seen my mom raising me since at a very young age, till now. In one way is more towards repaying, like helping her to like repaying her gratitude. It's like a gratitude that sort of thing. So that's why I feel that you know to take care of grandma as well as taking care of my mom as well..." (Samuel)<br><br>"This past year I just recently lived in a different environment and lived with my husband's uncle who has intellectual disability. I got to know about his situation like few years back when he started living with my in-laws. So before that I roughly got to know like, what, how he would like sometimes act out...to the extent of I know la that the situation in the household... before this I I didn't have any family member who had any like intellectual disability or any physical disability for that matter. So, I never had to help out as much as I do now in this my current household situation. So, this is you will have to volunteer yourself more because I'm a daughter-in-law. So, YOU as a daughter-in-law also have responsibility to help out mother-in-law [primary caregiver]…" (Farhana) |

| | | |
|---|---|---|
| | 1.2 Transition by circumstance: *“I was put into this situation.”* | "So this [caregiving] is actually due to my family situation. My parents got divorced. None of my parents actually wanted to take responsibility of me and my older sister...we ended up staying with my paternal grandparents...I think the first one is it's it doesn't sit well with me is because, like I was kind of PUT into this situation.…" (Xiahui)<br><br>" we suffered a domestic abuse from our parents so that led to us shifting out...[to grandmother’s residence]…I would say that the primary cause of me being caregiver for anybody right is that it just became the set of circumstances just led it to develop very organically..." (Ryan)<br><br>"my mom got uhh.. diagnosed with brain tumor last year after her Hari Raya. And yeah, that was how I first got into caregiving...I just assumed that responsibility, cause. I thought like, it was supposed to be my duty anyway…because I'm the oldest and I have to take care of my three younger siblings, basically being the breadwinner… I mean, since I was I mean the first child." (Siti) |

| | | |
|---|---|---|
| 2. Grappling with role conflicts and expectations: *"I'm still trying to find my balance."* | 2.1 Amidst competing demands: a conflicted and constrained life | "I'm STILL TRYING TO find my balance. You know, trying to improve myself as a wife and as a daughter-in-law who is capable of managing the household at the same time having a full-time job...(sigh)...I have to help out once I return from the office...during at night, I prepare his [care recipient] supper. Yeah, so sometimes you know sometimes after the day in the office, you get very tired. So you want to rest, but then you know there's things to do around the house." (Farhana)<br><br>"Actually we have intentions of renting out a flat so that you know, while waiting for our house to be ready, like it will take a few years so, but actually, because of this [caregiving] situation, we actually made the decision to just stay and be there for her [mother-in-law primary caregiver]." (Farhana)<br><br>"I can't expect so much from my husband ah…So you know, like I will cheap out on certain things, you know? Because I know that he has to provide to help his mother [primary caregiver] out to provide for his uncle [care recipient] as well. So, I won't like ask for anything extravagant things you know...I know that he is trying uh, a lot, trying a lot. I mean he's trying his best to provide financially for his mother as well as his uncle. So I have to understand..." (Farhana)<br><br>"I don't know if they have like high subsidy for like hiring maids, you know…she [mother-in-law primary caregiver] keeps telling us that she wants a maid but currently we don't, we are not like in uhh.. in the financial situation to provide her maid right now. So yeah, that's something that me and my husband have to deal with lah." (Farhana)<br><br>"I started weighing it out very logically, with the amount of school work that I have now, and with the need to start earning some money to be able to provide for the household la. I do not reasonably have the time for CCAs as well, because at that point of time it is either I don't starve and I have money and I'm working or I have good grades, but I have lesser money or I can forsake both of these and then have CCAs...because there's only so many hours in a week that I can work while not compromising my caregiving. Yeah, there's always only so many I can put into work right without compromising school and caregiving" (Ryan)<br><br>"I would say it was VERY CHALLENGING also in the sense that I had to juggle so many things at once. Yeah, and at the same time like prioritizing my mum health...caregiving was SO time-consuming that I really didn't have time for almost anything else, with my friends or my boyfriend or even for myself...over |

| | | |
|---|---|---|
| | | time I felt like I was burning out. I was REALLY VERY tired and I I myself was starting to show I was starting to show symptoms like having headaches and having all kinds of back pain, body pain (titter) everything else. Yeah, and there were a few times where I also had a fever because I was caring for her...I felt physically weaker after caregiving and, I felt like I wasn't really my best...when I came to school or like work and all that yeah. Uh, yeah, it was an uncomfortable pain" (Siti) |

| | | |
|---|---|---|
| 2. Grappling with role conflicts and expectations: *"I'm still trying to find my balance."* | 2.1 Amidst competing demands: a conflicted and constrained life | "So, uh, I realized that, uhm, any anything any social thing would really DRAIN my energy SO MUCH and I think it is because I really spending so much time caregiving and like uh, spending emotional labor with my mom...that made me felt, that made me feel very uh… alone also after awhile...I didn't have as much time to devote to my CCAs my school activities or like gatherings with my classmates…" (Siti)<br><br>"let's say [care recipient wants] a piece of cake or anything. But the thing is that I'm actually quite busy right now so I couldn't get it for her [care recipient]. I'm busy with my projects discussions and having lessons in school. So I tried to talk to her in terms of like you know, I currently busy so I couldn't get it for her. But she just die die wants it now la!" (Samuel)<br><br>"Like in terms of socializing with my friends will be restricted in some ways...As well as the money to be spend will be limited as well. Because need to buy groceries as well." (Samuel)<br><br>"...the demands of caregiving is too high, it can really affect my social life can affect my work. It can also affect my performance because you know, just suddenly taking urgent leave you know. It's not something that a lot of bosses will accept. and I feel I actually have done that quite a few times" (Xiahui)<br><br>"it's because it's my first year [at workplace], and like its my first career...My performance worse or something, they might appraise me. I don't know. Because everyone else is not really taking that many [leaves] so. And the first year, people expect you to grind to always want to learn to have a lot of determination and stuff. So maybe. Yeah, part of me also feels that I'll be left behind..." (Xiahui)<br><br>"You know it's very hard to just be productive all the time. Right? I really want to relax sometimes, spend some time by myself, but then it's hard to also have the social life. It's hard to, you know, maintain my hobbies. Like there are some things I want to do, but I can't really do because most of my time is taken up." (Xiahui)<br><br>"I think #1 would be financially. Money again, sorry. Yeah yeah, especially 'cause I'm just like fresh graduate. So, it's very hard for me to to contribute… Interviewer: I see. OK and if you had that financial support, how do you think it will help you? Xiahui: I think emotionally, I will be in a better place. I don't have to worry about so many different things at once because I'm worrying about, I mean, I'm still single. I'm living my grandma. I just paid off my student debt. So there's a lot of financial worry that I have and |

| | | |
|---|---|---|
| | | like sometimes like another form of money that I am giving is like my grandma's pocket money and I actually pay for the Grab to like the Polyclinic and stuff like that so. I mean, it adds up." (Xiahui)<br><br>"you as a working adult, you can't afford to, sometimes when you go home also I mean you're tired and then after that sometimes you fell asleep already. Then after that, these people (care recipient) only get to COME OUT of the house because it depends on whether YOU'RE FREE or not...So it IS SAD to see that la" (Imran) |

| | | |
|---|---|---|
| 2. Grappling with role conflicts and expectations: *"I'm still trying to find my balance."* | 2.2 Perceived inadequacy amidst role identity and expectations | "just feeling like I'm not good enough daughter…I'm not caring for her [care recipient] enough. So it's just feeling inadequate...I had a lot of self-doubts. I had these expectations on myself that I should be doing MORE..." (Siti)<br><br>"I also remember feeling like uh… I I wasn't doing enough for her. I had a lot of self-doubts...I felt like I had, I had these expectations on myself that I I should be doing MORE, even though it's just waking up every 15 minutes, 30 minutes to do all this. I shouldn't be complaining I should, you know, JUST DO yeah. And just do everything I can to make her feel comfortable and be able to recover faster...pressure, self-pressure that I put it on myself. Yeah 'cause also I wanted my mom to get the best, you know so… " (Siti)<br><br>"sometimes you will just reflect upon like "have you done your best? Have you tried your best" that kind of thing..." (Samuel)<br><br>"it turned out that my grandma nearly tripped again. So that's where the worry came back again, uh, because do I really want my grandma to actually have a second incident that happened towards her that kind of thing...Yeah, yeah, to be honest, it's quite awful. Haha.. The the feeling is quite awful ah.. because. I am a person that tend to be much more responsible kind of person. So… if something nearly fails means that I actually failed as a grandchild of not taking care of her already. Yeah, so it's like, if something actually being compromised... I will definitely feel that, I did not take care of her truly well enough, and that's where burdens have to be carried throughout my whole entire life." (Samuel)<br><br>"… as much as I'm I'm mostly trying my best so that you know, she you know to be good enough for her [mother-in-law]. I mean sometimes it gets VERY stressful...as a daughter-in-law, I know my mother-in-law is, her personality, she's quite critical. So uhm, she's very, uhm, I would say OCD about things in the house. So sometimes you know, it's uhm, very hard to be ENOUGH, uhm, to be a good enough daughter-in-law." (Farhana) |

| | | |
|---|---|---|
| | 2.2 Perceived inadequacy amidst role identity and expectations | **[NOTE: Contrary to other participants, Xiahui derives inadequacy in her caregiving role from the perceived lack of reciprocity and affection from the care recipient]**<br><br>"ALWAYS ME like helping her [care recipient], but I understand she's old so. Yeah, like there's no value for me . It's just I guess the only value is us maintaining that "positive relationship", "positive relationship" [Xiahui gestured air quotes]. It is always about her needs and wants. Besides forcing myself to think feel good because her needs and wants are fulfilled, what is in it for me?" (Xiahui)<br><br>"It's a very ONE-SIDED kind of relationship...I am actually quite protective of myself. So that's where sometimes quarrelling can happen, 'cause I don't really like giving, giving and giving. But in caregiving that's, you know, as the name suggests caregiving right? There is a lot of giving expected, yeah." (Xiahui)<br><br>"...caregiving for my grandma has taken a toll on my self-esteem. Yeah, I think it's also, add on to the fact that she keeps reminding me that I am unwanted. And that I should be very grateful to her so, that's one of the challenge. I'm just always disgruntled but I do it [caregiving] anyway." (Xiahui) |

| | | |
|---|---|---|
| 3. Navigating intergenerational dynamics and relational distress | 3.1 Resentment over a lack of support from the middle generation | "my grandma has FOUR children, but none of them are really taking responsibility, and one of them is my dad, who is not only not taking responsibility of his CHILDREN, but his mum as well. So that…to put it bluntly, it PISSES ME OFF! So yeah, that's it. [Xiahui expresses frustration whilst stroking her hair]...So there were a lot of times where I had to go around asking people [middle generation family members to assist with care recipient's medical appointments]… Then in the end everyone cannot make it that kind of thing." (Xiahui)<br><br>"I DON'T SEE a logic in there whereby you know he is your blood related. You need to, you know he is your blood-related and it's a person with disabilities, SPECIAL disabilities. So to me is NONSENSE for them to give money and then after that say "Yaa just take this money and take care of him" [Imran]<br><br>"I mean, they are not caring for him. Why are they [extended family middle generation] talking SO MUCH?" [Farhana]<br><br>"Be it my cousin or even my aunt's side. It's like they don't see our...they don't see me and my mum's [primary caregiver] difficulty. But they only say like "eh, she's [care recipient] fine leh". To them "she is fine leh. She's not like acting [behavioural challenges of care recipient] like...". But WE are the ones that actually live together with her. We DEFINITELY.. WE ARE THE ONES that first-hand experience." [Samuel]<br><br>" you can't change, you CAN'T FORCE person [extended family] who don't really have this intention of taking care of her. It's like you're forcing someone to eat something that is they REALLY dislike. That kind of thing ah." [Samuel]<br><br>"my father is out of the picture. The eldest daughter of my grandparents is also out of the picture. So, its left with my grandparents' youngest daughter and their first-born grandson [Ryan] which was actually capable of providing care...my parents were estranged from my grandparents, actually. Uh, and then my eldest aunt is actually a bit delusional and hysterical. I would say...she has some form of, some onset of a mental problem already...So rationally right, it [caregiving] will just fall onto the two most sanest persons of the house.." [Ryan] |

| | | |
|---|---|---|
| 3. Navigating intergenerational dynamics and relational distress | 3.2 Sense of agency in shouldering care | "I always told her [primary caregiver] this. If they [extended family] ask, tell them that "My SON has money. So I can ask from my son". I wasn't quite happy with my mother's side...I fought for my mother, I fought for my mother, back then and I fought for her. I didn't want her to go through all this SHIT again and to go through all this MESS again to see this MESS being repeated was a hassle la and then to see your own mother getting (sigh). You know the feeling when you see your own mother getting scolded by her brothers and sisters...." (Imran)<br><br>"I felt that it was "CRAZY" because I was directly dealing with a life or death situation for my mum [care recipient], and that it was overwhelming because of the conflict of opinions among my [extended] family members...So like I just felt like I should just step up [as a daughter]. And just like you know, you should just go on with the surgery" (Siti)<br><br>"I'm the oldest and I have to take care of my three younger siblings. So I, uh, I gave pocket money to my younger sister who's in Poly and then helping to fork out a bit more money for groceries. Yeah, and just basically being the breadwinner in that point in time, yeah...I was the key decision maker now, like I had to ensure that my family had enough food at home." (Siti)<br><br>"I've taken on more of the caregiving role from [Aunt's] plate so that she doesn't have to concern herself with that much..."because I I was able to ease into the role organically by my own initiative, right? There were more changes I did organically" (Ryan)<br><br>"I feel that me and my mom [primary caregiver] will actually rather take care of her [care recipient] rather than my cousin's wise. Yeah, because you know we know how to take care of, but they they [extended family] are the ones that don't really have a first-hand experience that kind of thing." (Samuel) |

| | | |
|---|---|---|
| 3. Navigating intergenerational dynamics and relational distress | 3.3 Distress from care recipient's behavioural challenges | "So the biasness towards me as well as my another two cousins...So yeah, it's like, she [care recipient] treat the two cousins WAY better than me la in I won't say in some ways, but in EVERY aspect....I kept questioning myself like…. uh…why, why, why am I, why am I taking care of her? Why is it me? Why not just call the the two cousins to actually take care of her instead? It will be much more easier. She'll probably have a better, HAPPIER LIFE LAH!" (Samuel)<br><br>"I tend to be frustrated at times because like can you [care recipient] like at least wait for a while to actually let me finish everything first before I do anything? To actually really go and settle her [care recipient] stuff and, but yeah, but in between have to really have to learn how to STOP whatever I'm doing just to help her out everything ah.. So yeah, it's kind of a FRUSTRATION" (Samuel)<br><br>"...when we took him [care recipient] in the early stage, I couldn't hold my temper also. I I need to treat him like a child….he will SHOUT at you back. Then when HE SHOUT at you back he SHOUTS at his loudest. So that is whereby, your part as your patience, your patience, they say patience is a virtue then after that you can see lah whether that patience is a virtue is real or not lah" (Imran) |

| | | |
|---|---|---|
| 3. Navigating intergenerational dynamics and relational distress | 3.3 Distress from care recipient's behavioural challenges | "certain times I do like, certain moments, I have to like scold him [care recipient], you know, because he will do things that you know he shouldn't be doing. And then I get a reaction out from HIM. And then I think once he actually hit me, because I think, I don't know whether he got shocked or he didn't like that I scolded him. It wasn't really like scolding. It was like, I said "don't do that" lah. That's all. And then he smacked me... So, uhm.. now I sometimes I do like try to tell him that something is wrong, but I can't do it so often and I have to be careful when I do it, because sometimes he gets VERY active and uhm, sometimes, you know, I'm very unbothered person. So if you know like when he hits me like, I try not to be affected by it…" (Farhana)<br><br>"sometimes you have to teach them the right way so, but then at the same time, you're AFRAID that he will lash out even more. So the balance between that is very tricky sometimes" (Farhana)<br><br>"the stress is escalated right is when making medical decisions because there's a prolonged interaction right, and trying to. You're trying to explain to her [care recipient] why this procedure is good for you, why this medication is good for you, and basically all I get is "no, I don't.". Then you are trying to like, "HOW!? " (Ryan)<br><br>"Yeah, because she [care recipient] doesn't want to live in a room with us [Ryan and siblings], right? And she refuses to leave the hole [hoarded environment]. So for a period of time she had a COVID back-to-back with dengue and I needed to provide care for her. I was I needed to provide medical assessment and I need to plan what was next right.. Going in was so difficult, right, I decided to ask my sisters to just throw things over the sofa. So I can just stay in there. Going in and out is so tiring...These sort of sights would be common to see in slums or whatnot. You just won't expect it to see in a living room. Yeah, you won't expect to see such a contraction in a living room in a HDB. It's the only thing that can't change." (Ryan) |

| | | |
|---|---|---|
| 3. Navigating intergenerational dynamics and relational distress | 3.3 Distress from care recipient's behavioural challenges | "the thing is like Grandma has like my grandma this child mindset rather.. So it tends to be very triggering as well...she doesn't really understand because her mental health state is like in her children young children that kind of mental state whereby, uh.. they feel another point of view. They will see in another point point of view rather than a normal human behavior's point of view." (Samuel)<br><br>"So kids will throw threw TANTRUM if they don't get the things that they want... They will cry out LOUD they will cry their LUNGS out loud, saying that they want that. It is the SAME thing. It is this the SAME thing. It's just in a different body...just treat him like because he's in the MIND of a five-year-old. So you need to TREAT him like a five year old person." (Imran)<br><br>"I think the main reason why I struggle with caregiving is more of the emotional aspect rather than the physical or any of like the resources that I have to give or sacrifice. I think that the one thing that makes caregiving very difficult for me is the thing that like is how my grandma [care recipient] reacts or how she pays back or pays me back for the amount [of caregiving] I've given to her like you know I mentioned, the very hurtful words and her trying to like to manipulate me into doing things by crying and her lack of respect for my choices...I think is very difficult because that's just how my grandma is yeah..." (Xiahui)<br><br>"I think something about my relationship with my grandma is that she kind of likes to rub it in that I'm very unfortunate. Yeah, she will say stuff like in Chinese [Mandarin] like "your mum and dad doesn't want you. You should be very lucky that Ah Ma", which is her, "takes care of you". So, I DON'T LIKE IT! But she's been saying this for years and years, and I have been telling her to stop, but it doesn't work…I mean no one likes hearing this kind of STUFF, I MEAN, I KNOW why do you have to keep repeating it? Like WHAT IS THAT GOING TO DO?" (Xiahui)<br><br>"I REALLY don't mind spending the time to [caregiving]. But, only when she [care recipient] doesn't do it in such a, very like agitated way or like overly emotional way I'm I'm really very open to help. And you know, spend time to explain to her or keep repeating the same thing over and over and over. I'm OK with it. But then like when she says those mean like when she says those mean things, and that's where I get very very frustrated, I get very hurt. And that's when I just really HATE caregiving." (Xiahui)<br><br>"she has this habit where she likes to. OK, I I don't know if it's really fake cry, but she [care recipient] likes to do that A LOT to get what she wants.... It FRUSTRATES ME A LOT.." (Xiahui) |

| | | |
|---|---|---|
| | | “in the middle of the night she [care recipient] just knocks on my door. And then she would tell me that she feels very sad. Then, I’ll just spend some time talking to her…last time in the past, quite often. She would just like start screaming [at night]. And then, she will tell me that she's quite concerned because she said she cannot help it… I get worried, like what is happening!? yeah…Well, actually like a lot of consoling and companionship at night” (Xiahui)<br><br>“My grandma tends to sleep like at the toilet like on the toilet bowl like in the middle of the night, she goes to the toilet. Then she'll just sit there and then she will fall asleep. Then when I wake up in the middle of the night, I get a shock. And sometimes I have to clean up after her in the middle of the night” (Xiahui).<br><br>"One thing that I wish that I had or can do is I can converse with her [care recipient] properly like can speak Mandarin well or she can speak English. Yeah, because sometimes I feel like my feelings cannot get through to her because I can't properly express myself. So, I end up having very high expectations and I get disappointed. But it's not really her fault." (Xiahui)<br><br>“I think there is a picture of a lot of medicine. Yeah, I guess that's like one of the struggles I have with caregiving. The WHOLE LOT of medicine and I think that I, we usually had quite a lot of quarrels because of the medications. Yeah so. I think like she depends on me, but also at the same time she also wants to be independent. So I was I was given this like really long, long list of medicine, of like when to take and dose and stuff like that. So I actually went to buy, you know, like those on Monday to Sunday kind of thing. Then nighttime morning time, how much to take and stuff like that and I like spend a lot of time reading and like sorting them out. Then my grandma [care recipient] refused to take them because she said she doesn't trust me. Yeah, and then, that really frustrated me. But then she keeps, she said she doesn't trust me. She thinks that I got it wrong, but then she keeps asking me like, “oh so. This, what what tablet is this?”. Then she would hand it to me. And then I get very frustrated. You don't trust me, but you trust me with that like I, I don't know. So that's one example with quarrelling.” (Xiahui)<br><br>"Because they are so used to doing things that they want right they, I would say that they will have a stronger conflicts between the identity and role confusion aspect of it. Because they're so used to not having their decisions questioned...They will experience that more than aging or anything else...Because of their very strong identity as I am myself, I do not need help being told what I need to do....it will |

| | | |
|---|---|---|
| | | definitely be a very strong source of conflict...ultimately as she still views me as the boy that wets the beds and does not eat chili. There were times that she doesn’t listen to me due to her insisting she knows better. however, due to my pursuit of the medical field for a good i would say 5 years at least she knows there is value to the things I say and doesn’t instantly dismiss them now." (Ryan)<br><br>"Yeah, because he [care recipient] doesn't know what he is doing (smiles), so he's just reacting like a child, yeah...So it's, certain things you have to like discipline him on like teaching a child....Sometimes, sometimes I do feel sad for him that he came into that circumstance without, you know he doesn't want to be ill, but then he is just uhmm, his brain, sometimes he would overthink and then he will lash out and then sometimes I know it aggravates other, the people around him but...you know that, he can't do anything about it, and then the more you, scold him, it will just act out more...we sometimes know that he's trying to communicate, you know what he is feeling but we do not understand him...The challenge is mostly when it gets very overwhelming when he acts when he is unhappy and then he, I don't know, we are unable to, you know, help him you know, feel better. " (Farhana) |
