# Supplementary figures and images for: Exploring the Lived Experiences of Caregiving for Older Family Members by Young Caregivers in Singapore: Transition, Trials, and Tribulations
Source: Int J Environ Res Public Health. 2024 Feb 5;21(2):182. doi: 10.3390/ijerph21020182 (PMC10888348; doi:10.3390/ijerph21020182)

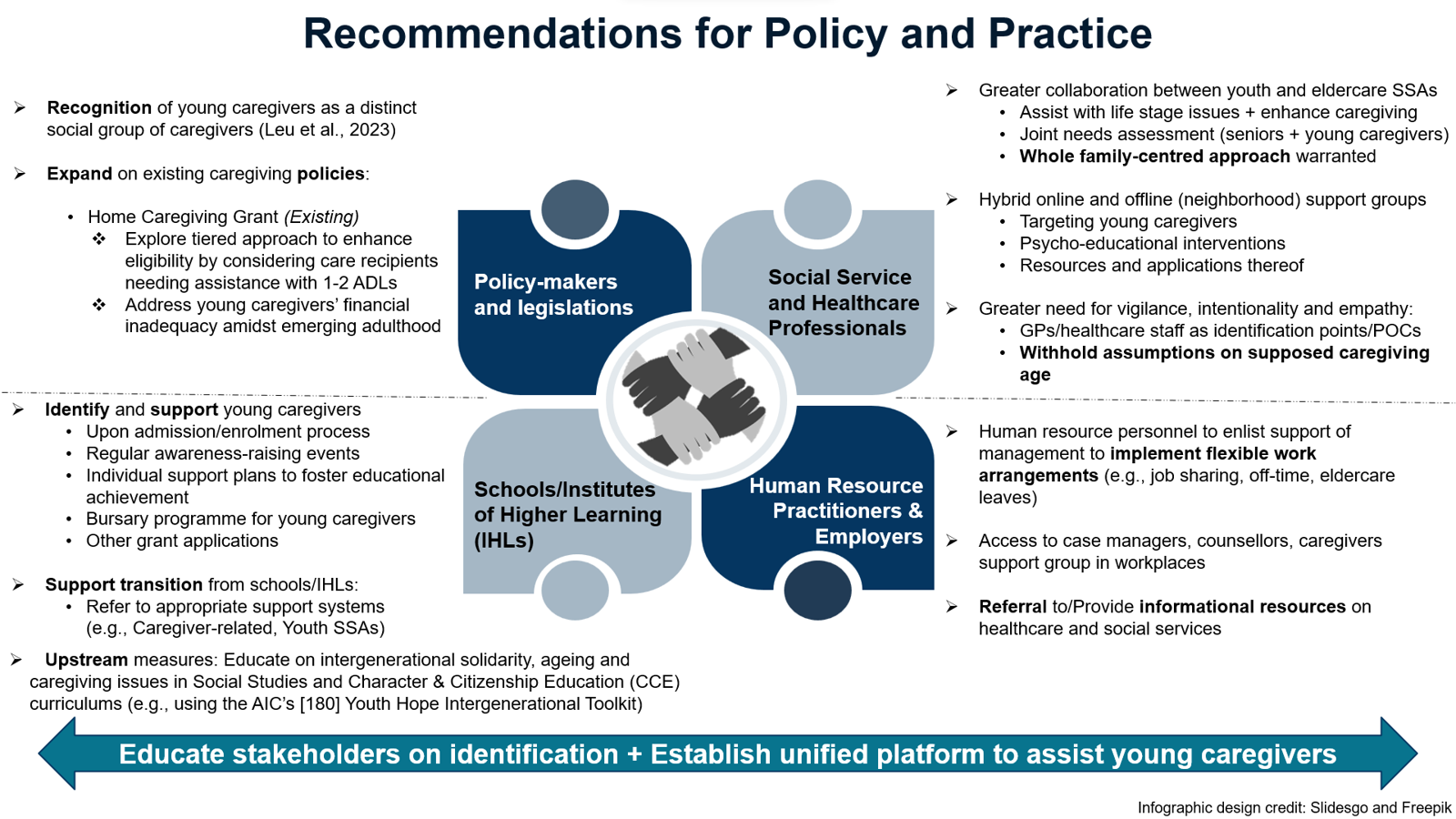

Supplement: Supplementary file 1 [file ijerph-21-00182-s001.zip › File S2 - Recommendations for Policy and Practice.png]
